# Clinical roles of *EGFR* amplification in diffuse gliomas: a real-world study using the 2021 WHO classification of CNS tumors

**DOI:** 10.3389/fnins.2024.1308627

**Published:** 2024-02-26

**Authors:** Hai Wang, Xin Zhang, Jiahui Liu, Wenlin Chen, Xiaopeng Guo, Yaning Wang, Yuekun Wang, Hao Xing, Tingyu Liang, Yixin Shi, Delin Liu, Tianrui Yang, Yu Xia, Junlin Li, Jiaming Wu, Qianshu Liu, Tian Qu, Siying Guo, Huanzhang Li, Kun Zhang, Yilin Li, Shanmu Jin, Dachun Zhao, Yu Wang, Wenbin Ma

**Affiliations:** ^1^Department of Neurosurgery, Center for Malignant Brain Tumors, National Glioma MDT Alliance, Peking Union Medical College Hospital, Chinese Academy of Medical Sciences and Peking Union Medical College, Beijing, China; ^2^China Anti-Cancer Association Specialty Committee of Glioma, Beijing, China; ^3^Eight-year Medical Doctor Program, Chinese Academy of Medical Sciences and Peking Union Medical College, Beijing, China; ^4^"4+4" Medical Doctor Program, Chinese Academy of Medical Sciences and Peking Union Medical College, Beijing, China; ^5^Department of Pathology, Peking Union Medical College Hospital, Chinese Academy of Medical Sciences and Peking Union Medical College, Beijing, China

**Keywords:** diffuse gliomas, 2021 WHO classification of central nervous system tumors, *EGFR* amplification, integrated diagnosis, glioblastoma

## Abstract

**Background:**

The 2021 World Health Organization Classification of Central Nervous System Tumors updates glioma subtyping and grading system, and incorporates *EGFR* amplification (Amp) as one of diagnostic markers for glioblastoma (GBM).

**Purpose:**

This study aimed to describe the frequency, clinical value and molecular correlation of *EGFR* Amp in diffuse gliomas based on the latest classification.

**Methods:**

We reviewed glioma patients between 2011 and 2022 at our hospital, and included 187 adult glioma patients with available tumor tissue for detection of *EGFR* Amp and other 59 molecular markers of interest. Clinical, radiological and pathological data was analyzed based on the status of *EGFR* Amp in different glioma subtypes.

**Results:**

163 gliomas were classified as adult-type diffuse gliomas, and the number of astrocytoma, oligodendroglioma and GBM was 41, 46, and 76. *EGFR* Amp was more common in IDH-wildtype diffuse gliomas (66.0%) and GBM (85.5%) than IDH-mutant diffuse gliomas (32.2%) and its subtypes (astrocytoma, 29.3%; oligodendroglioma, 34.8%). *EGFR* Amp did not stratify overall survival (OS) in IDH-mutant diffuse gliomas and astrocytoma, while was significantly associated with poorer OS in IDH-wildtype diffuse gliomas, histologic grade 2 and 3 IDH-wildtype diffuse astrocytic gliomas and GBM.

**Conclusion:**

Our study validated *EGFR* Amp as a diagnostic marker for GBM and still a useful predictor for shortened OS in this group.

## Introduction

The most prevalent primary malignant brain tumors are gliomas, most of which grow invasively and lack a clear boundary with normal brain tissue, and hence are defined as diffuse gliomas. Despite a low average annual incidence of about 8 per 100,000, gliomas have a grave prognosis, with a 5-year survival rate of 6.9% for the most aggressive subtype of glioblastoma (GBM; Ostrom et al., 2022). Gliomas are characterized by various histopathology and genetic heterogeneity. In pursuit of more precise glioma diagnosis and prognostic prediction, the World Health Organization (WHO) Classification of Central Nervous System (CNS) Tumors has been updated to the fifth edition (WHO CNS5) in 2021 (Louis et al., 2021). Compared with the WHO CNS4 classification in 2016 which mostly relies on tumor histology (Louis et al., 2016), the latest version makes major changes in the categorization scheme of gliomas and incorporates several molecular markers related to different subtypes. Currently, diffuse gliomas are divided into adult-type, pediatric-type low-grade and pediatric-type high-grade. Adult-type diffuse gliomas, as the predominant pathological type, include astrocytoma, IDH-mutant; oligodendroglioma, IDH-mutant and 1p/19q-codeleted; and glioblastoma, IDH-wildtype.

**Figure 1:**
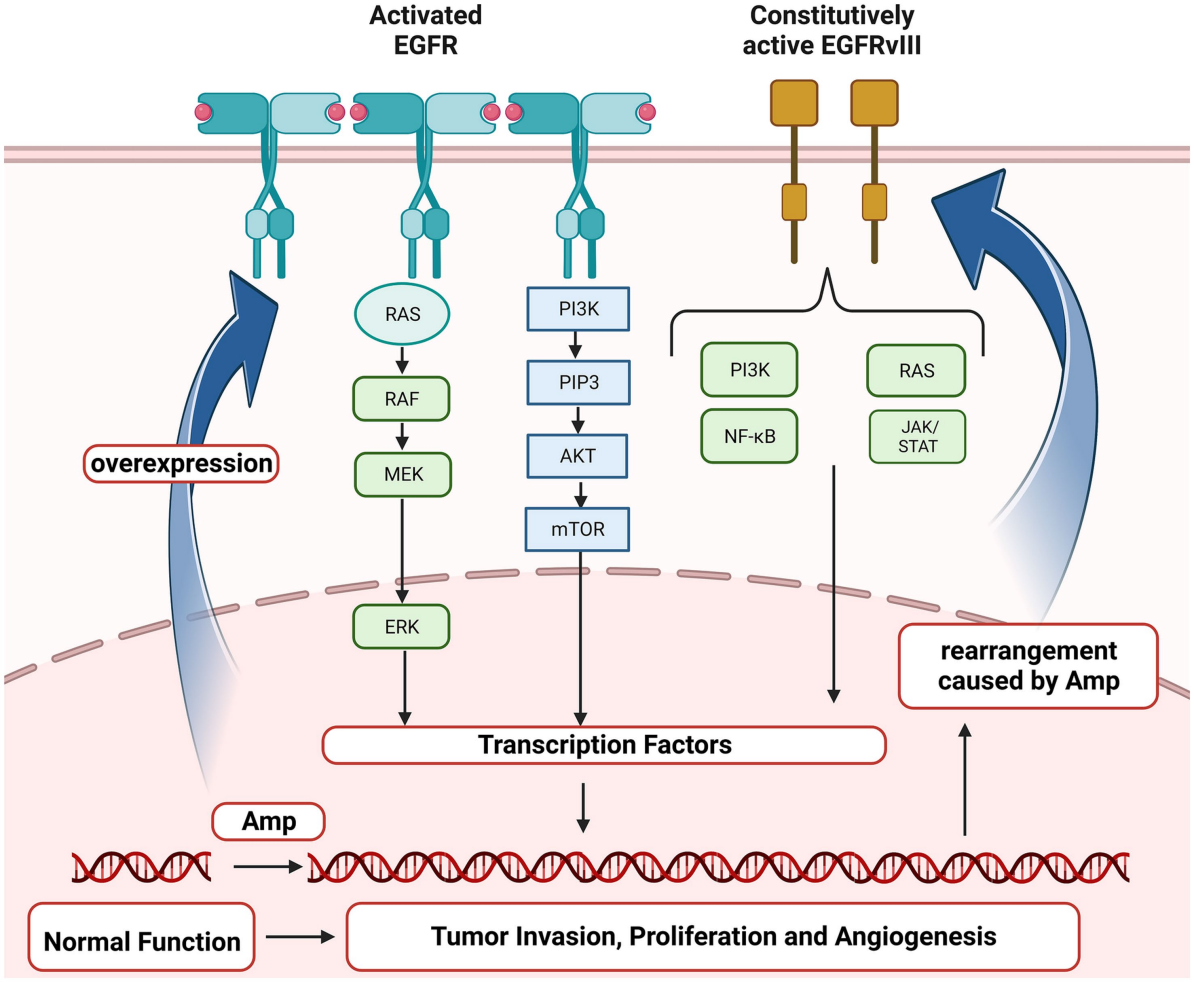
Oncogenic *EGFR* signaling pathway in glioma. *EGFR*, Epidermal growth factor receptor; *EGFRvIII*, Epidermal growth factor receptor variant III; RAS, a family of genes (HRAS, KRAS and NRAS) involved in signaling pathways of cell growth and death; RAF, Serine/threonine-protein kinases; MEK, Mitogen-activated protein kinase; ERK, Extracellular signal-regulated kinases; PI3K, Phosphoinositide 3-kinase; PIP3, Phosphatidylinositol (3,4,5)-trisphosphate; AKT, Protein kinase B; mTOR, mammalian target of rapamycin; NF-κB, Nuclear factor kappa-light-chain-enhancer of activated B cells; JAK/STAT, Janus kinase/signal transducers and activators of transcription; Amp, amplification.

Epidermal growth factor receptor (*EGFR*) gene, located on the short arm of human chromosome 7 (chr 7p11.2), encodes a transmembrane glycoprotein that is a member of receptor tyrosine kinases (RTKs) and regulates cell proliferation (Voldborg et al., 1997). *EGFR* amplification (Amp) and mutations have been identified as driving events for multiple cancers, non-small cell lung cancer (NSCLC), breast cancer and GBM in particular (da Cunha et al., 2011; Brennan et al., 2013; Hsu and Hung, 2016; Sigismund et al., 2018). *EGFR* Amp was reported to occur in nearly two-thirds of primary GBM, and almost half of those positive for *EGFR* Amp harbored the mutant *EGFRvIII* and *EGFR* single nucleotide variants (SNVs; Brennan et al., 2013; Furnari et al., 2015; Eskilsson et al., 2018; Munoz-Hidalgo et al., 2020). As shown in Figure 1, *EGFR* Amp leads to overexpression of EGFR protein in glioma cells, contributing to tumor proliferation, angiogenesis and invasion via RAS and PI3K/AKT signaling pathway. Besides, especially in GBM, genomic rearrangement caused by *EGFR* Amp increases the occurrence of *EGFRvIII*, which could activate PI3K/AKT and other downstream pathways independent of extracellular ligand, exerting pro-tumorigenic effects (Yang et al., 2017). Studies in recent years have found that the prognosis of IDH-wildtype diffuse lower grade gliomas with *EGFR* Amp overlapped with that of GBM (Aibaidula et al., 2017; Stichel et al., 2018; Petersen et al., 2021). Therefore, the WHO CNS5 classification has added *EGFR* Amp as a key molecular marker for diagnosing IDH-wildtype GBM in the absence of microvascular proliferation (MVP) and necrosis (Louis et al., 2021). However, *EGFR* Amp was less investigated in other diffuse gliomas, particularly those of IDH-mutant. It remains unknown about the landscape of *EGFR* Amp in diffuse gliomas under the current classification and whether it has new clinical roles in IDH-mutant diffuse gliomas. Therefore, it is necessary to restudy the frequency and prognostic value of *EGFR* Amp in different subtypes of diffuse gliomas defined by the WHO CNS5 classification.

This study enrolled 187 adult patients diagnosed with diffuse gliomas from January 2011 to April 2022 at our hospital and reclassified them according to the present WHO classification. We analyzed the frequency of *EGFR* Amp, and its association with patient prognosis and other genetic alterations in different subgroups, aiming to offer more knowledge about the application of *EGFR* Amp in the 2021 WHO classification.

## Materials and methods

### Patient population

605 patients with glioma, resected or biopsied between January 2011 and January 2022 at the Department of Neurosurgery at Peking Union Medical College Hospital (PUMCH), were screened for this study (Guo et al., 2023). Exclusion criteria were: (i) age under 18 years; (ii) missing data for diagnostic molecular markers (i.e., *IDH1/2* mutation, *CDKN2A/B* mutation, 1p/19q codeletion, *TERT* promoter mutation, *EGFR* amplification and combined whole chromosome 7 gain and whole chromosome 10 loss); (iii) circumscribed astrocytic gliomas, glioneuronal and neuronal tumors and ependymal tumors according to the 2021 WHO classification of CNS tumors. 187 patients were included for further analysis. This study was approved by the Institutional Ethics Review Board of PUMCH (ID: S-424), and all participants provided written informed consent.

### Data collection

Clinical information of all patients was collected from the medical records, including gender, age at diagnosis, body mass index (BMI), disease duration before admission, baseline Karnofsky Performance Score (KPS), primary or recurrent tumors, clinical symptoms, the extent of resection (EOR) and postoperative treatments. The survival status was collected via outpatient and telephone follow-ups. The overall survival (OS) was defined as the time from operation to the patient’s death or last follow-up (censored).

Baseline MRI images of all patients were retrieved from the Picture Archiving and Communication System (PACS) to extract radiological features, including number of tumors, tumor locations, involvement of eloquent areas, signal intensity on T1WI and T2WI, presence of contrast enhancement, peritumoral edema and necrotic center, and maximal diameter of tumor, edema and necrosis. Two junior neuroradiologists evaluated specific features separately, and one neuroradiologist with over-10-year working experience examined the results.

Histopathological data were acquired from the pathological reports by the department of pathology at PUMCH, including Ki-67 index and histological WHO grade. Formalin-fixed, paraffin-embedded tumor tissue sections were subjected to next-generation sequencing (NGS), polymerase chain reaction (PCR)-based assays, and fluorescence *in situ* hybridization methods (FISH) for the detection of 60 molecular markers (Supplementary Table S1). These markers were chosen based on recent studies on tumorigenesis and prognosis of glioma. DNA was extracted with QIAGEN 56404 Kit, and DNA concentration and purity were determined by Qubit 4.0 Fluorometer (Thermo Fisher Scientific) and Nanodrop 2000 spectrophotometer (Thermo Fisher Scientific) respectively. After DNA fragmentation and PCR amplification, double-ended sequencing was performed by NovaSeq 6000. Copy number variation was identified from DNA sequencing results using CNVkit. The objective criteria for deletion were the ratio of copy number ≤ 1.5 while the number of bin ≥ 0.3, and the objective criteria for amplification were the ratio of copy number ≥ 2.5 while the number of bin ≥ 0.3. FISH was applied to verify EGFR amplification, with the EGFR probe to chromosome 7 probe ratio ≥ 2.0. Histopathological and molecular pathological data were integrated to determine the subtypes of gliomas according to the 2021 WHO classification of CNS tumors.

### Statistical analyses

Continuous variables were presented as the mean ± standard deviation (SD) or median plus interquartile range (IQR) based on data distribution, while categorial variables were presented as number plus percentage. Each variable was compared between *EGFR* amplification (Amp) and non-amplification (Non-amp) in different subtypes. Normally distributed continuous variables were compared by Student’s *t*-test, while non-normally distributed continuous variables were compared by Mann–Whitney *U* test. The comparison of categorical variables was performed using the chi-squared test. The difference of OS between *EGFR* Amp and Non-amp in different groups was evaluated with the Kaplan–Meier method and the log-rank test. Besides, Fisher’s exact test was performed to analyze the correlation between *EGFR* Amp and other genes’ alteration, and the results were illustrated by the heatmap of -1og10(*p*-value). *p* < 0.05 was considered as statistically significantly for all statistical analyses. SPSS (version 26.0, IBM, United States) statistical software and R software (version 4.2.1) were used for data analysis, and GraphPad Prism (9, GraphPad Software, United States) software was used for graphic drawing.

## Results

### Subtyping of diffuse gliomas using the WHO CNS5 classification and frequency of *EGFR* amplification

**Figure 2:**
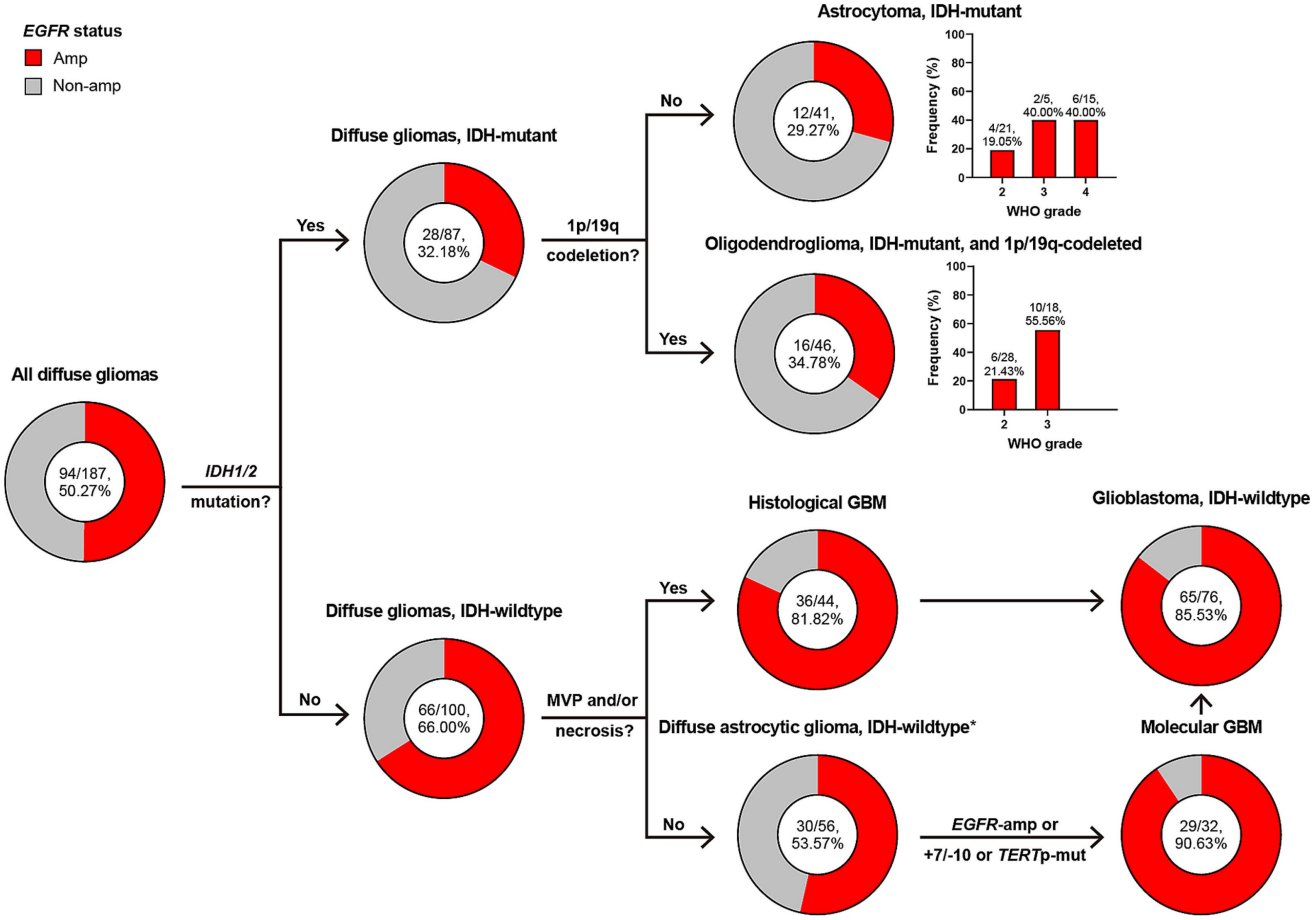
Classification of diffuse gliomas according to histological and molecular markers, and frequency of *EGFR* amplification in different subgroups. The total patient number, and the number plus percentage of patients with *EGFR* amplification in each group are presented. *Diffuse astrocytic glioma, IDH-wildtype refers to histologic grade 2 and 3 IDH-wildtype diffuse astrocytic gliomas, as is stated in cIMPACT-NOW update 3 (Brat et al., 2018). Amp, amplification; Non-amp, non-amplification; MVP, microvascular proliferation; GBM, glioblastoma, IDH-wildtype; +7/−10, combined whole chromosome 7 gain and whole chromosome 10 loss; *TERT*p, *TERT* promoter; Mut, mutation.

Figure 2 illustrates the subtyping flow of 187 diffuse gliomas with intact diagnostic molecular markers. Initially, they were screened for *IDH1/2* mutation, and divided into 87 IDH-mutant and 100 IDH-wildtype diffuse gliomas. Of 87 IDH-mutant diffuse gliomas, 41 astrocytoma, IDH-mutant and 46 oligodendroglioma, IDH-mutant and 1p/19q-codeleted were confirmed based on the absence or presence of 1p/19q-codeletion. 44 of 100 IDH-wildtype diffuse gliomas presented microvascular proliferation (MVP) or necrosis and were defined as histological glioblastoma (GBM). 56 of 100 IDH-wildtype diffuse gliomas without MVP or necrosis were defined as diffuse astrocytic glioma, IDH-wildtype, and further screened for *EGFR* amplification (Amp), +7/−10 and *TERT* promotor mutation, with 32 of them defined as molecular GBM. 44 histological and 32 molecular GBM made up the group of glioblastoma, IDH-wildtype under the WHO CNS5 classification.

*EGFR* Amp appeared in 50% of all diffuse gliomas, but was more common in those of IDH-wildtype (66% vs. 32%). Astrocytoma and oligodendroglioma shared similar frequency of *EGFR* Amp (29% and 34%), with higher in WHO grade 3 or 4. The presence of *EGFR* Amp was consistent in histological, molecular and all GBM, with a frequency of 81%, 90%, and 85%, respectively.

### Clinical, radiological, and pathological features based on *EGFR* amplification and non-amplification

Baseline information of three types of adult-type diffuse gliomas, namely astrocytoma, oligodendroglioma and GBM, was detailed in Table 1. Clinical, radiological and histopathological differences between *EGFR* Amp and Non-amp were explored. Less GBM patients with *EGFR* Amp developed symptoms of intracranial hypertension (headache and/or vomiting; 40.0% vs. 90.9%). Oligodendroglioma with *EGFR* Amp tended to have larger maximal tumor diameter (5.83 ± 1.81 vs. 4.30 ± 2.13). Besides, oligodendroglioma with *EGFR* Amp was more likely to manifest as higher histological grade.

**Table 1:** Clinical, radiological, and pathological characteristics of adult-type diffuse gliomas based on *EGFR* status.

Subtype	Astrocytoma, IDH-mutant		Oligodendroglioma, IDH-mutant and 1p/19q-codeleted		Glioblastoma, IDH-wildtype	
*EGFR* status	Amp	Non-amp	Amp	Non-amp	Amp	Non-amp
Number of patients	12	29	16	30	65	11
*Basic information*						
Male	10 (83.3%)	18 (62.1%)	8 (50.0%)	23 (76.7%)	46 (70.8%)	5 (45.5%)
Mean age (years)	40.42 ± 9.55	40.66 ± 10.48	43.94 ± 9.77	43.37 ± 10.58	56.89 ± 13.51	48.09 ± 19.87
Mean BMI (kg/m^2^)	23.97 ± 2.68	24.38 ± 3.12^†^	24.67 ± 3.38	25.51 ± 3.72	24.05 ± 2.87^†^	22.45 ± 3.44
Median disease duration (weeks)	5 (3.25, 11)	4 (2.65, 16)	8 (5, 90)	5.5 (2.08, 52.5)	4 (2, 12)	4 (1.5, 16)
Median baseline KPS	85 (80, 90)	90 (80, 90)	90 (80, 90)*	90 (90, 90)*	80 (72.5, 90)	90 (60, 90)
Primary	12 (100%)	23 (79.3%)	14 (87.5%)	27 (90.0%)	54 (83.1%)	9 (81.8%)
Intracranial hypertension^a^	9 (75.0%)	13 (44.8%)	9 (56.3%)	10 (33.3%)	26 (40.0%)*	10 (90.9%)*
Neurologic impairment	8 (66.7%)	23 (79.3%)	14 (87.5%)	21 (70.0%)	53 (81.5%)	9 (81.8%)
Epilepsy	4 (33.3%)	15 (51.7%)	7 (43.8%)	17 (56.7%)	25 (38.5%)	1 (9.1%)
*Radiological features*						
**Lesion number**						
Single	9 (75.0%)	26 (89.7%)	14 (87.5%)	29 (100%)	50 (76.9%)	9 (81.8%)
Multiple	2 (16.7%)	1 (3.4%)	2 (12.5%)	0 (0.0%)	9 (13.8%)	1 (9.1%)
NA	1 (8.3%)	2 (6.9%)	-	1 (3.3%)	6 (9.2%)	1 (9.1%)
**Lesion side**						
Left	5 (41.7%)	9 (31.0%)	8 (50.0%)	16 (53.3%)	33 (50.8%)	4 (36.4%)
Right	4 (33.3%)	14 (48.3%)	5 (31.3%)	12 (40.0%)	22 (33.8%)	6 (54.5%)
Bilateral	2 (16.7%)	4 (13.8%)	3 (18.8%)	1 (3.3%)	4 (6.2%)	0 (0.0%)
NA	1 (8.3%)	2 (6.9%)	-	1 (3.3%)	6 (9.2%)	1 (9.1%)
**Lesion location**						
Single lobe	7 (58.3%)	13 (44.8%)	10 (62.5%)	19 (63.3%)	29 (44.6%)	4 (36.4%)
Multiple lobes	0 (0.0%)	8 (27.6%)	1 (6.3%)	6 (20.0%)	19 (29.2%)	4 (36.4%)
Cross midline structures	4 (33.3%)	6 (20.7%)	5 (31.3%)	4 (13.3%)	11 (16.9%)	2 (18.2%)
NA	1 (8.3%)	2 (6.9%)	-	1 (3.3%)	6 (9.2%)	1 (9.1%)
**Involvement of eloquent areas**						
Yes	2 (16.7%)	9 (31.0%)	3 (18.8%)	5 (16.7%)	28 (43.1%)	3 (27.3%)
No	9 (75.0%)	17 (58.6%)	13 (81.3%)	24 (80.0%)	27 (41.5%)	6 (54.5%)
NA	1 (8.3%)	3 (10.3%)	-	1 (3.3%)	10 (15.4%)	2 (18.2%)
**T1WI signal intensity**						
Low	6 (50.0%)	15 (51.7%)	10 (62.5%)	25 (83.3%)	19 (29.2%)	2 (18.2%)
Mixed	4 (33.3%)	10 (34.5%)	5 (31.3%)	4 (13.3%)	36 (55.4%)	7 (63.6%)
NA	2 (16.7%)	4 (13.8%)	1 (6.3%)	1 (3.3%)	10 (15.4%)	2 (18.2%)
**T2WI signal intensity**						
High	5 (41.7%)	11 (37.9%)	5 (31.3%)	17 (56.7%)	19 (29.2%)	2 (18.2%)
Mixed	5 (41.7%)	14 (48.3%)	10 (62.5%)	12 (40.0%)	36 (55.4%)	7 (63.6%)
NA	2 (16.7%)	4 (13.8%)	1 (6.3%)	1 (3.3%)	10 (15.4%)	2 (18.2%)
**Contrast enhancement**						
Yes	3 (25.0%)	10 (34.5%)	7 (43.8%)	12 (40.0%)	51 (78.5%)	8 (72.7%)
No	7 (58.3%)	14 (48.3%)	8 (50.0%)	16 (53.3%)	3 (4.6%)	1 (9.1%)
NA	2 (16.7%)	5 (17.2%)	1 (6.3%)	2 (6.7%)	11 (16.9%)	2 (18.2%)
**Peritumoral edema**						
Yes	3 (25.0%)	14 (48.3%)	9 (56.3%)	16 (53.3%)	48 (73.8%)	8 (72.7%)
No	7 (58.3%)	11 (37.9%)	6 (37.5%)	12 (40.0%)	7 (10.8%)	1 (9.1%)
NA	2 (16.7%)	4 (13.8%)	1 (6.3%)	2 (6.7%)	10 (15.4%)	2 (18.2%)
**Necrotic center**						
Yes	3 (25.0%)	12 (41.4%)	8 (50.0%)	12 (40.0%)	45 (69.2%)	8 (72.7%)
No	7 (58.3%)	13 (44.8%)	7 (43.8%)	16 (53.3%)	7 (10.8%)	1 (9.1%)
NA	2 (16.7%)	4 (13.8%)	1 (6.3%)	2 (6.7%)	13 (20.0%)	2 (18.2%)
Tumor maximum diameter (cm)	4.74 ± 1.41^†^	4.63 ± 1.62^†^	5.83 ± 1.81^†*^	4.30 ± 2.13^†*^	3.84 ± 1.68^†^	4.51 ± 1.45^†^
Edema maximum diameter (cm)	2.03 ± 1.55	1.83 ± 0.79^†^	2.06 ± 1.33^†^	1.73 ± 0.71	2.54 ± 1.48^†^	2.61 ± 1.21
Necrosis maximum diameter (cm)	2.23 ± 1.07	2.69 ± 0.87^†^	3.02 ± 1.05^†^	1.96 ± 1.28	2.57 ± 1.38^†^	2.89 ± 1.63
*Treatment*						
**Extent of resection**						
Total	7 (58.3%)	14 (48.3%)	12 (75.0%)	22 (73.3%)	39 (60.0%)	7 (63.6%)
Subtotal	2 (16.7%)	4 (13.8%)	0 (0.0%)	3 (10.0%)	2 (3.1%)	2 (18.2%)
Partial	1 (8.3%)	8 (27.6%)	1 (6.3%)	2 (6.7%)	13 (20.0%)	0 (0.0%)
Biopsy	2 (16.7%)	3 (10.3%)	3 (18.8%)	3 (10.0%)	11 (16.9%)	2 (18.2%)
**Postoperative treatment**						
Radiotherapy	3 (25.0%)	3 (10.3%)	1 (6.3%)	2 (6.7%)	3 (4.6%)	0 (0.0%)
TMZ-based chemotherapy	1 (8.3%)	3 (10.3%)	2 (12.5%)	6 (20.0%)	10 (15.4%)	2 (18.2%)
TMZ-based chemoradiotherapy	6 (50.0%)	15 (51.7%)	9 (56.3%)	14 (46.7%)	28 (43.1%)	1 (9.1%)
None	0 (0.0%)	1 (3.4%)	0 (0.0%)	0 (0.0%)	5 (7.7%)	0 (0.0%)
NA	2 (16.7%)	8 (27.6%)	4 (25.0%)	8 (26.7%)	19 (29.2%)	8 (72.7%)
*Pathological data*						
**Histological grade**						
WHO grade 2	6 (50.0%)	21 (72.4%)	6 (37.5%)^*^	22 (73.3%)^*^	9 (13.8%)	1 (9.1%)
WHO grade 3	5 (41.7%)	7 (24.1%)	8 (50.0%)^*^	8 (26.7%)^*^	20 (30.8%)	2 (18.2%)
WHO grade 4	1 (8.3%)	1 (3.4%)	2 (12.5%)^*^	0 (0.0%)^*^	36 (55.4%)	8 (72.7%)
Median Ki-67 (%)	8 (3, 30) ^†^	5 (3, 10) ^†^	10 (5, 30) ^†^	5 (3, 15) ^†^	30 (10, 50)^†^	22.5 (4.5, 30) ^†^

Data are presented with *n* (%), Mean ± SD or Median (IQR). Some categorial variables do not add up to 100% due to missing values. Incomplete continuous variables are marked with †, all of which have ≤4 missing values except 12 in the “tumor maximum diameter” of “Glioblastoma, IDH-wildtype” with EGFR amplification. Comparisons of each variable are made between EGFR Amp and Non-amp in three different subtypes separately. ^*^p<0.05. ^a^Symptoms of intracranial hypertension referred to headache and/or vomiting. Amp, amplification; Non-amp, non-amplification; NA, not available; BMI, body mass index; KPS, Karnofsky Performance Score; TMZ, temozolomide.

Baseline information of other IDH-wildtype diffuse gliomas was detailed in Supplementary Table S2. Since there was only *EGFR* Amp or Non-amp in each subtype, no comparison was made.

### Overall survival differences between *EGFR* amplification and non-amplification in different subtypes of diffuse gliomas

**Figure 3:**
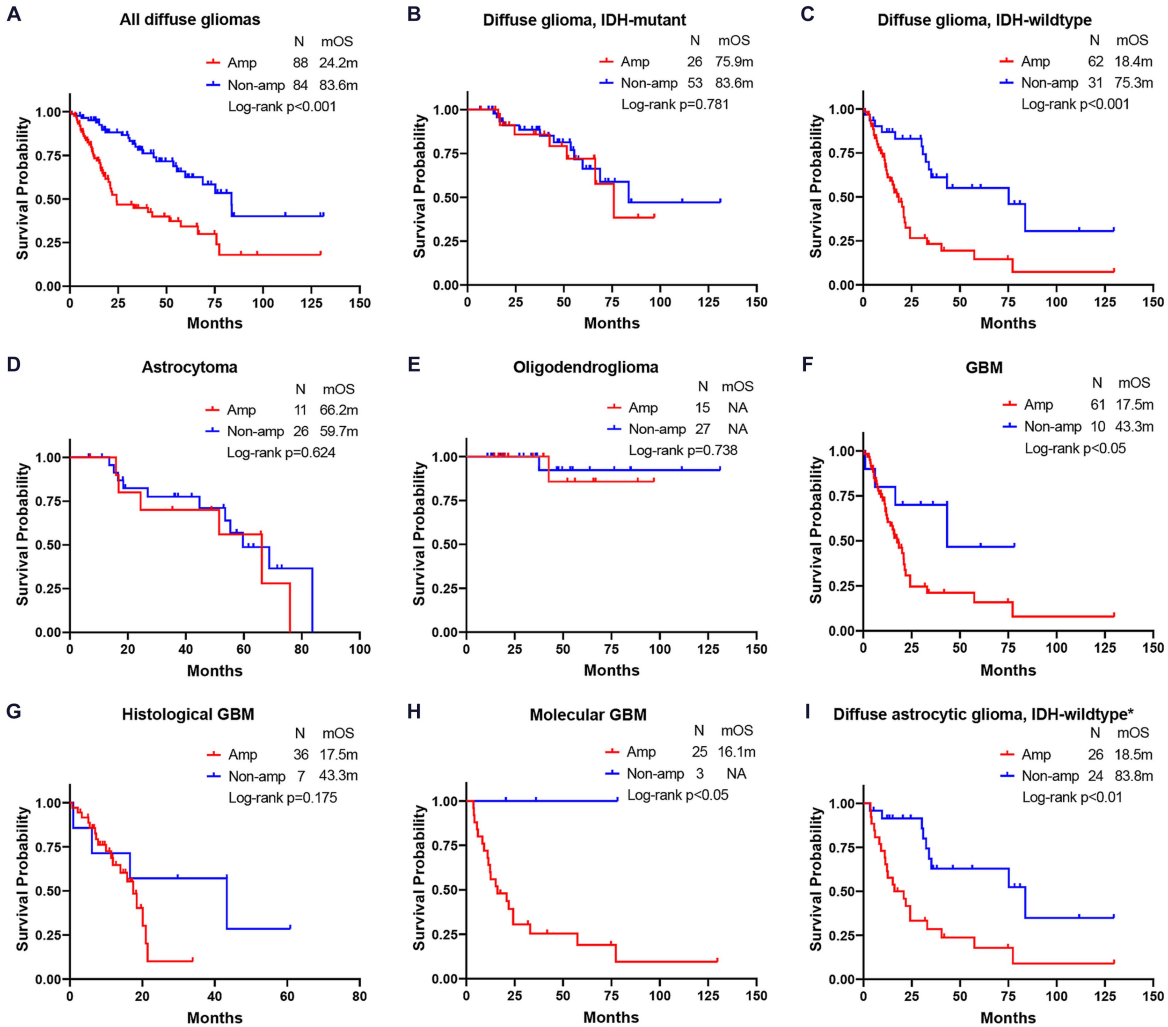
Comparison of overall survival between *EGFR* amplification and non-amplification in different subgroups of diffuse gliomas. **(A)** all diffuse gliomas; **(B)** diffuse glioma, IDH-mutant; **(C)** diffuse glioma, IDH-wildtype; **(D)** astrocytoma; **(E)** oligodendroglioma; **(F)** glioblastoma (GBM); **(G)** histological GBM; **(H)** molecular GBM; **(I)** diffuse astrocytic glioma, IDH-wildtype. The patient number, mOS and Log-rank *p*-value are presented. *Diffuse astrocytic glioma, IDH-wildtype refers to histologic grade 2 and 3 IDH-wildtype diffuse astrocytic gliomas, as is stated in cIMPACT-NOW update 3 (Brat et al., 2018). Amp, amplification; Non-amp, non-amplification; NA, not available; mOS, median overall survival.

*EGFR* Amp manifested as an unfavorable molecular marker for median overall survival (mOS) in all diffuse gliomas [24.2 months vs. 83.6 months, hazard ratio (HR) = 2.76, *p* < 0.001] and IDH-wildtype diffuse glioma (18.4 months vs. 75.3 months, HR = 2.94, *p* < 0.001), while not for IDH-mutant diffuse glioma (75.9 months vs. 83.6 months, HR = 1.14, *p* = 0.781) and astrocytoma (66.2 months vs. 59.7 months, HR = 1.28, *p* = 0.624). *EGFR* Amp seemed not to discriminate mOS in astrocytoma of different WHO grades, either (Supplementary Figure S1). Histological GBM with *EGFR* Amp tended to have a shorter mOS than those of *EGFR* Non-amp (17.5 months vs. 43.3 months, HR = 1.93, *p* = 0.175). The mOS of both histologic grade 2 and 3 IDH-wildtype diffuse astrocytic gliomas and molecular GBM was significantly stratified by *EGFR* Amp (18.5 months vs. 83.8 months, HR = 3.30, *p* < 0.01, 16.1 months vs. NA, *p* = 0.044). Taken histological and molecular GBM together, namely GBM under the WHO CNS5 classification, *EGFR* Amp was still associated with significantly worse survival, with a mOS of 17.5 months (HR = 2.75, *p* = 0.039; Figure 3).

### Correlations between *EGFR* amplification and other genes’ alterations

**Figure 4:**
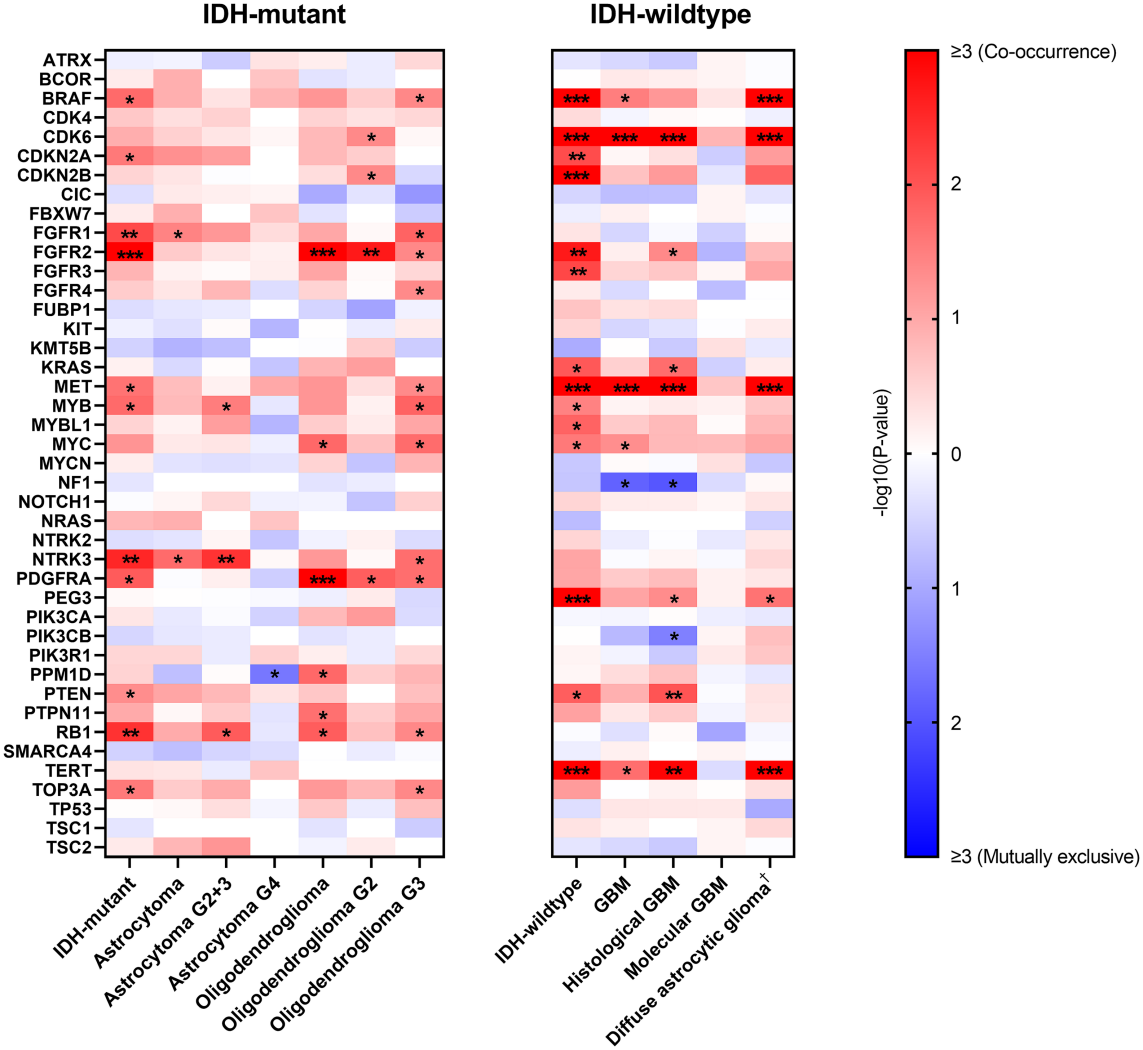
Correlations between *EGFR* amplification and other genes’ alterations in IDH-mutant and IDH-wildtype diffuse gliomas. −log10(*p*-value) is calculated to show correlation between paired genes, with red and blue indicating co-occurrence and mutually exclusive, respectively. Only genes with at least one correlation result are shown here (*ACVR1*, *HIST1H3B*, *HIST1H3C*, *H3F3A*, *MAP2K1*, *SMARCB1*, *YAP1* excluded) and white indicates no computed result for specific gene pairs. ^*^p < 0.05; ^**^p < 0.01; ^***^p < 0.001. †Diffuse astrocytic glioma, IDH-wildtype refers to histologic grade 2 and 3 IDH-wildtype diffuse astrocytic gliomas, as is stated in cIMPACT-NOW update 3 (Brat et al., 2018). G2, WHO grade 2; G3, WHO grade 3; G2 + 3, WHO grade 2 and 3; G4, WHO grade 4; GBM, glioblastoma, IDH-wildtype.

Given the prognostic value of *EGFR* Amp and in order to show the patterns of alterations in other genes, we analyzed the correlations between *EGFR* Amp and a set of selected genes in IDH-mutant and IDH-wildtype diffuse glioma and their subtypes (Figure 4).

In IDH-mutant diffuse glioma, *EGFR* Amp tended to co-occur with *FGFR1*, *FGFR2*, *NTRK3* and *RB1* alterations. This result was not completely consistent in astrocytoma and oligodendroglioma. However, IDH-wildtype diffuse glioma had a distinct pattern, with *BRAF*, *CDK6*, *CDKN2A/B*, *FGFR2*, *FGFR3*, *MET*, *PEG3* and *TERT* alterations more likely co-occurring with *EGFR* Amp. The co-occurrence of *CDK6* and *MET* alterations with *EGFR* Amp was consistent in all GBM, histological GBM and histologic grade 2 and 3 IDH-wildtype diffuse astrocytic gliomas, whereas no possibly correlated genetic alterations were found in molecular GBM.

## Discussion

In this study, we investigated the distribution of *EGFR* amplification (Amp) in different subtypes of diffuse gliomas based on the WHO CNS5 classification (Louis et al., 2021), its value for prognosis, and its relationship with other genetic changes. We found that *EGFR* Amp mainly occurred in IDH-wildtype diffuse gliomas, accounting for 66%, which was twice as high as that in IDH-mutant diffuse gliomas. In IDH-mutant diffuse gliomas, *EGFR* Amp tended to indicate higher WHO grade. In IDH-wildtype diffuse gliomas, *EGFR* Amp was mostly distributed in GBM, particularly molecular GBM. Additionally, *EGFR* Amp was linked to significantly worsened prognosis in all diffuse gliomas, IDH-wildtype diffuse gliomas, GBM, molecular GBM, and histologic grade 2 and 3 IDH-wildtype diffuse astrocytic gliomas. This result was consistent with the role of *EGFR* Amp in diffuse glioma in the WHO CNS5 classification (Louis et al., 2021). Finally, the correlation between *EGFR* Amp and other molecular alterations was found various among different subgroups and grades of diffuse gliomas.

The frequency of *EGFR* Amp was seldom depicted in IDH-mutant diffuse gliomas. Several previous studies included IDH-mutant GBM which is defined as IDH-mutant astrocytoma (WHO grade 4) currently, and examined *EGFR* Amp, with the ratio ranging from 3% to 16% (Verhaak et al., 2010; Brennan et al., 2013; Li et al., 2019; Wong et al., 2021; Umphlett et al., 2022). Besides, a study by Bai et al. found that 16 out of 86 (18%) IDH-mutant grade 2 to 3 gliomas were *EGFR*-amplified (Bai et al., 2016). However, description of *EGFR* Amp in oligodendroglioma is rare, with only one study in 2001 reporting a frequency of 31% in anaplastic oligodendrogliomas (WHO grade III) (Hoang–Xuan et al., 2001). Our study demonstrated that *EGFR* Amp was present in 29% of astrocytoma and 34% of oligodendroglioma, with higher frequency in WHO grade 3 or 4. The clinical recognition of *EGFR* Amp in our IDH-mutant glioma patients was close to previous reports, and the slightly higher frequency may be accounted for by the inclusion of *EGFR* gene in routine molecular tests for glioma since the publication of cIMPACT-NOW update 3 (Brat et al., 2018). As for GBM, 85% had amplified *EGFR* gene, with elevated ratio in molecular ones, reaching over 90%. These results were in line with the finding that *EGFR* Amp was more prevalent in IDH-wildtype gliomas, especially GBM (Brennan et al., 2013). Higher distribution of *EGFR* Amp in our GBM patients was due to the addition of IDH-wildtype lower grade astrocytoma with amplified-*EGFR* into the GBM of the WHO CNS5 classification.

We explored clinical, radiological, and pathological differences between *EGFR* Amp and Non-amp and most of comparisons yielded no significant difference. In oligodendroglioma, *EGFR*-amplified tumor had larger maximal tumor diameter. Although few articles reported similar finding, it could be explained by higher histological grade in *EGFR*-amplified oligodendroglioma in our study. In addition, less *EGFR*-amplified GBM patients developed symptoms of intracranial hypertension (headache and/or vomiting), but there were no differences in maximal tumor and edema diameter. Such discrepancy may be related to missing radiological data in our research. Therefore, further verification through complete clinical information is necessary.

*EGFR*, as an oncogenic gene, has been extensively investigated for its prognostic value in gliomas, especially IDH-wildtype gliomas. *EGFR* Amp has been established as an independent marker for poor overall survival (OS) in IDH-wildtype lower grade gliomas, which was close to that of GBM (Aibaidula et al., 2017; Stichel et al., 2018; Petersen et al., 2021). Our study verified *EGFR* Amp as an unfavorable marker for OS in IDH-wildtype histologic grade 2 and 3 astrocytic gliomas, with a median OS (mOS) of 18.5 months. This result indicated the accuracy of the 2021 WHO classification of CNS tumors. Besides, we also implied that *EGFR* Amp was potentially meaningful in all GBM and histological GBM, although the difference in the latter group was not statistically significant. Two recent studies on prognostic molecular markers in GBM yielded different results about *EGFR*. In the retrospective study by Sirui Ma et al., univariable analysis of 367 adult patients with IDH-wildtype GBM (both histological and molecular) showed that *EGFR* Amp was not significantly associated with OS (Ma et al., 2020). In the study by Peter H. Yang et al., *EGFR* mutation was associated with decreased OS in the subset analysis of 167 patients with IDH-wildtype GBM (Yang et al., 2022). However, Peter H. Yang et al.’s study was based on the 2016 WHO criteria and did not reveal the specific result on *EGFR* Amp (Yang et al., 2022). Therefore, the prognostic role of *EGFR* Amp in the new entity of GBM under the 2021 WHO criteria needs further investigation. On the contrary, in IDH-mutant gliomas, *EGFR* alterations were less common and its prognostic value was under evaluated (Bai et al., 2016; Umphlett et al., 2022). A study by Craig Horbinski et al. used *EGFR* immunohistochemistry (IHC) and showed that *EGFR* expression failed to discriminate survival among astrocytic tumors (Horbinski et al., 2011). Additionally, strong *EGFR* expression was associated with reduced survival in WHO grade II oligodendrogliomas, but was a favorable marker for survival in WHO grade III anaplastic oligodendrogliomas (Horbinski et al., 2011). A recent study suggested that *EGFR* Amp in WHO grade 4 IDH-mutant astrocytoma was not related to worse OS, unless *CDKN2A/B* homozygous deletion were also detected (Li et al., 2019). In our study, OS did not differ significantly among astrocytoma and its different WHO grades. Meanwhile, owing to insufficient endpoints, we were unable to determine the role of *EGFR* Amp in predicting oligodendroglioma patients’ OS. Thus, further exploration of *EGFR* Amp in IDH-mutant gliomas should be considered in tumors of specific grade or together with other genetic alterations.

Furthermore, we calculated the correlations between *EGFR* Amp and other genetic changes. In previous studies, oligodendroglioma was found associated with *CIC* and *FUBP1* due to the close chromosomal location of these genes and 1p/19q co-deletion (Sahm et al., 2012; Yip et al., 2012), while astrocytoma mostly presented *TP53* and *ATRX* mutations (Cancer Genome Atlas Research Network et al., 2015). In our cohort of IDH-mutant diffuse glioma, *EGFR* Amp was found to co-occur with *FGFR1*, *FGFR2*, *NTRK3* and *RB1* alterations, which was not completely consistent in astrocytoma and oligodendroglioma. These results corresponded to the comparatively low frequency of *EGFR* Amp in IDH-mutant diffuse glioma and its subtypes. Besides, given the relatively small number of IDH-mutant glioma patients in our cohort, these co-occurrence results need to be validated in larger group. In IDH-wildtype diffuse glioma, a different pattern was seen, with *BRAF*, *CDK6*, *CDKN2A/B*, *FGFR2*, *FGFR3*, *MET*, *PEG3* and *TERT* alterations most co-occurring with *EGFR* Amp. *EGFR* Amp co-occurred with *CDK6* and *MET* alterations in all GBM, histological GBM and histologic grade 2 and 3 IDH-wildtype diffuse astrocytic gliomas. These genes have been reported to link with tumorigenesis and progression of GBM, especially *CDK6*, *CDKN2A/B*, *MET* and *TERT* (Xu and Li, 2018; Cheng and Guo, 2019). Nevertheless, in molecular GBM, *EGFR* Amp did not have significantly correlated genetic alterations. Such difference in molecular pattern between histological GBM and molecular GBM suggested that *EGFR*-amplified GBM with or without histological malignancy is different in oncogenesis. Further researches should be conducted to explore the value of these biomarkers.

However, our study results must be interpreted while considering some limitations. Firstly, statistical analyses were only conducted in patients with intact molecular data, and thus selection bias should be taken into account. Secondly, our patient cohort was not large enough and the follow-up of some subgroups (e.g., oligodendroglioma) was not long enough, which potentially interfere with the analysis. Thirdly, forms of molecular alteration are various, in which simply classifying *EGFR* and other genes as amplified and non-amplified or normal and altered may cover up some meaningful changes. Besides, our panel was pre-designed with certain molecular markers, meaning that more molecular correlations may not be accounted for.

## Conclusion

In this real-world study of 187 adult patients, we described the frequency of *EGFR* Amp, and explored its clinical, radiological and pathological characteristics in diffuse gliomas under the 2021 WHO classification of CNS tumors. *EGFR* Amp was confirmed as a significant prognostic biomarker for all IDH-wildtype diffuse glioma, histologic grade 2 and 3 IDH-wildtype diffuse astrocytic gliomas and GBM, but a limited one for IDH-mutant diffuse glioma and its subtypes. However, molecular correlations indicated that further classification may be required for some types. Our findings further verified the clinical implications of *EGFR* Amp in diffuse gliomas, and suggested future research should be undertaken on its association with other molecular alterations to offer more precise diagnosis, treatment and prognostic prediction of glioma.

## Data availability statement

The original contributions presented in the study are included in the article/Supplementary material, further inquiries can be directed to the corresponding authors.

## Ethics statement

The studies involving humans were approved by Institutional Review Board of Peking Union Medical College Hospital (S-424). The studies were conducted in accordance with the local legislation and institutional requirements. The participants provided their written informed consent to participate in this study.

## Author contributions

HW: Data curation, Formal Analysis, Writing – original draft, Writing – review & editing. XZ: Data curation, Formal Analysis, Writing – original draft, Writing – review & editing. JiL: Writing – review & editing. WC: Conceptualization, Data curation, Writing – review & editing. XG: Conceptualization, Data curation, Writing – review & editing. YanW: Data curation, Writing – review & editing. YueW: Data curation, Writing – review & editing. HX: Data curation, Writing – review & editing. TL: Data curation, Writing – review & editing. YS: Data curation, Writing – review & editing. DL: Data curation, Writing – review & editing. TY: Data curation, Writing – review & editing. YX: Data curation, Writing – review & editing. JuL: Data curation, Writing – review & editing. JW: Data curation, Writing – review & editing. QL: Data curation, Writing – review & editing. TQ: Data curation, Writing – review & editing. SG: Data curation, Writing – review & editing. HL: Data curation, Writing – review & editing. KZ: Data curation, Writing – review & editing. YL: Data curation, Writing – review & editing. SJ: Data curation, Writing – review & editing. DZ: Data curation, Writing – review & editing. YuW: Funding acquisition, Supervision, Writing – review & editing. WM: Funding acquisition, Supervision, Writing – review & editing.

## Supplementary material

SUPPLEMENTARY FIGURE S1 Comparison of overall survival between *EGFR* amplification and non-amplification in different grades of astrocytoma. The patient number, mOS and Log-rank p value are presented. G2+3, WHO grade 2 and 3; G4, WHO grade 4; Amp, amplification; Non-amp, non-amplification; mOS, median overall survival.
