# Supplementary figures and images for: Clinical roles of EGFR amplification in diffuse gliomas: a real-world study using the 2021 WHO classification of CNS tumors
Source: Front Neurosci. 2024 Feb 26;18:1308627. doi: 10.3389/fnins.2024.1308627 (PMC11002900; doi:10.3389/fnins.2024.1308627)

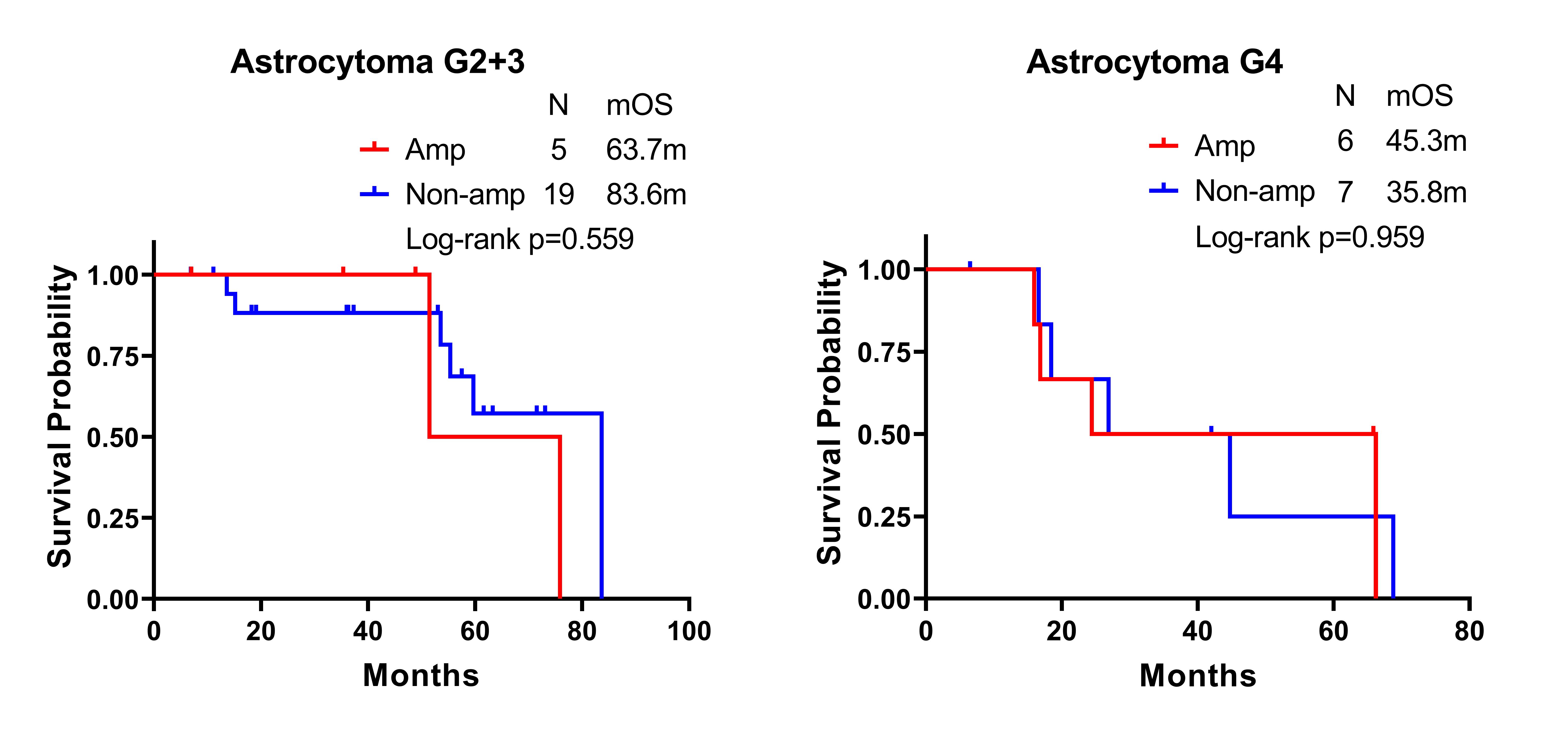

Supplement: SUPPLEMENTARY FIGURE S1 — Comparison of overall survival between EGFR amplification and non-amplification in different grades of astrocytoma. The patient number, mOS and Log-rank p value are presented. G2+3, WHO grade 2 and 3; G4, WHO grade 4; Amp, amplification; Non-amp, non-amplification; mOS, median overall survival. [file Figure_1.tif]
