# Supplementary material for: Clinical roles of EGFR amplification in diffuse gliomas: a real-world study using the 2021 WHO classification of CNS tumors
Source: Front Neurosci. 2024 Feb 26;18:1308627. doi: 10.3389/fnins.2024.1308627 (PMC11002900; doi:10.3389/fnins.2024.1308627)
Supplement: Supplementary file 2 [file Table_1.docx]

**Table S1.** The complete detection list of molecular markers.

| Detection List of Molecular Markers | | | | | | | | | |
| --- | --- | --- | --- | --- | --- | --- | --- | --- | --- |
| ACVR1 | ATRX | BCOR | BRAF | CDK4 | CDK6 | CDKN2A | CDKN2B | chr10p | chr10q |
| chr17 | chr19q | chr1p | chr7p | chr7q | chr9p | CIC | EGFR | FBXW7 | FGFR1 |
| FGFR2 | FGFR3 | FGFR4 | FUBP1 | H3F3A | HIST1H3B | HIST1H3C | IDH1 | IDH2 | KIT |
| KMT5B | KRAS | MAP2K1 | MET | MYB | MYBL1 | MYC | MYCN | NF1 | NOTCH1 |
| NRAS | NTRK2 | NTRK3 | PDGFRA | PEG3 | PIK3CA | PIK3CB | PIK3R1 | PPM1D | PTEN |
| PTPN11 | RB1 | SMARCA4 | SMARCB1 | TERT | TOP3A | TP53 | TSC1 | TSC2 | YAP1 |
