# Supplementary material for: Clinical roles of EGFR amplification in diffuse gliomas: a real-world study using the 2021 WHO classification of CNS tumors
Source: Front Neurosci. 2024 Feb 26;18:1308627. doi: 10.3389/fnins.2024.1308627 (PMC11002900; doi:10.3389/fnins.2024.1308627)
Supplement: Supplementary file 3 [file Table_2.docx]

**Table S2.** Clinical, radiological, and pathological characteristics of other IDH-wildtype diffuse gliomas.

| **Subtype** | Diffuse pediatric-type high-grade glioma, H3-wildtype and IDH-wildtype | Diffuse glioma, IDH-wildtype, NEC | Diffuse astrocytoma, *MYB*- or *MYBL1*-altered | Diffuse midline glioma, H3 K27-altered |
| --- | --- | --- | --- | --- |
| ***EGFR* status** | Non-amp | Non-amp | Non-amp | Amp |
| **Number of patients** | 10 | 7 | 6 | 1 |
| *Basic information* |  |  |  |  |
| **Male** | 6 (60.0%) | 4 (57.1%) | 4 (66.7%) | 1 (100%) |
| **Mean age (years)** | 48.10±11.76 | 42.43±11.79 | 39.29±16.80 | 45 |
| **Mean BMI (kg/m^2^)** | 23.45±2.51 | 26.57±4.68 | 23.52±2.47 | 20.42 |
| **Median disease duration (weeks)** | 18.5 (6.25, 34) | 16 (4, 104) | 13 (3, 24) | 3 |
| **Median baseline KPS** | 85 (80, 90) | 90 (80, 90) | 90 (70, 90) | 70 |
| **Primary** | 9 (90.0%) | 5 (71.4%) | 5 (83.3%) | 1 (100%) |
| **Intracranial hypertension**^a^ | 5 (50.0%) | 3 (42.9%) | 3 (50.0%) | 0 (0.0%) |
| **Neurologic impairment** | 8 (80.0%) | 6 (85.7%) | 4 (66.7%) | 1 (100%) |
| **Epilepsy** | 1 (10.0%) | 3 (42.9%) | 1 (16.7%) | 0 (0.0%) |
| *Radiological features* |  |  |  |  |
| **Lesion number** |  |  |  |  |
| Single | 4 (40.0%) | 5 (71.4%) | 5 (83.3%) | 1 (100%) |
| Multiple | 2 (20.0%) | 2 (28.6%) | 1 (16.7%) | 0 (0.0%) |
| **Lesion side** |  |  |  |  |
| Left | 2 (20.0%) | 4 (57.1%) | 5 (83.3%) | 1 (100%) |
| Right | 3 (30.0%) | 2 (28.6%) | 0 (0.0%) | 0 (0.0%) |
| Bilateral | 1 (10.0%) | 1 (14.3%) | 1 (16.7%) | 0 (0.0%) |
| **Lesion location** |  |  |  |  |
| Single lobe | 1 (10.0%) | 3 (42.9%) | 3 (50.0%) | 0 (0.0%) |
| Multiple lobes | 1 (10.0%) | 1 (14.3%) | 1 (16.7%) | 0 (0.0%) |
| Cross midline structures | 4 (40.0%) | 3 (42.9%) | 2 (33.3%) | 1 (100%) |
| **Involvement of eloquent areas** |  |  |  |  |
| Yes | 1 (10.0%) | 0 (0.0%) | 4 (66.7%) | 0 (0.0%) |
| No | 4 (40.0%) | 7 (100%) | 2 (33.3%) | 1 (100%) |
| **T1WI signal intensity** |  |  |  |  |
| Low | 4 (40.0%) | 4 (57.1%) | 5 (83.3%) | 1 (100%) |
| Mixed | 2 (20.0%) | 3 (42.9%) | 1 (16.7%) | 0 (0.0%) |
| **T2WI signal intensity** |  |  |  |  |
| Equal | 0 (0.0%) | 0 (0.0%) | 1 (16.7%) | 0 (0.0%) |
| High | 4 (40.0%) | 4 (57.1%) | 4 (66.7%) | 1 (100%) |
| Mixed | 2 (20.0%) | 3 (42.9%) | 1 (16.7%) | 0 (0.0%) |
| **Contrast enhancement** |  |  |  |  |
| Yes | 5 (50.0%) | 4 (57.1%) | 3 (50.0%) | 1 (100%) |
| No | 1 (10.0%) | 3 (42.9%) | 3 (50.0%) | 0 (0.0%) |
| **Peritumoral edema** |  |  |  |  |
| Yes | 6 (60.0%) | 4 (57.1%) | 3 (50.0%) | 0 (0.0%) |
| No | 0 (0.0%) | 3 (42.9%) | 3 (50.0%) | 1 (100%) |
| **Necrotic center** |  |  |  |  |
| Yes | 4 (40.0%) | 2 (28.6%) | 1 (16.7%) | 0 (0.0%) |
| No | 2 (20.0%) | 5 (71.4%) | 5 (83.3%) | 1 (100%) |
| **Tumor maximum diameter (cm)** | 4.47±2.65^†^ | 3.00±1.36 | 3.81±2.00 | 2.90 |
| **Edema maximum diameter (cm)** | 2.44±1.56^†^ | 3.87±3.09 | 3.39±2.24 | - |
| **Necrosis maximum diameter (cm)** | 2.16±1.35 | 2.51±1.57 | 4.99 | - |
| *Treatment* |  |  |  |  |
| **Extent of resection** |  |  |  |  |
| Total | 6 (60.0%) | 4 (57.1%) | 3 (50.0%) | 1 (100%) |
| Subtotal | 1 (10.0%) | 0 (0.0%) | 1 (16.7%) | 0 (0.0%) |
| Partial | 0 (0.0%) | 1 (14.3%) | 0 (0.0%) | 0 (0.0%) |
| Biopsy | 3 (30.0%) | 2 (28.6%) | 2 (33.3%) | 0 (0.0%) |
| **Postoperative treatment** |  |  |  |  |
| Radiotherapy | 0 (0.0%) | 1 (14.3%) | 0 (0.0%) | 0 (0.0%) |
| TMZ-based chemotherapy | 0 (0.0%) | 0 (0.0%) | 1 (16.7%) | 0 (0.0%) |
| TMZ-based chemoradiotherapy | 5 (50.0%) | 1 (14.3%) | 1 (16.7%) | 0 (0.0%) |
| Others | 1 (10.0%)^b^ | 0 (0.0%) | 0 (0.0%) | 0 (0.0%) |
| None | 0 (0.0%) | 0 (0.0%) | 0 (0.0%) | 0 (0.0%) |
| *Pathological data* |  |  |  |  |
| **Histological grade** |  |  |  |  |
| WHO grade 2 | 0 (0.0%) | 6 (85.7%) | 6 (100%) | 0 (0.0%) |
| WHO grade 3 | 10, 10% | 1 (14.3%) | 0 (0.0%) | 1 (100%) |
| WHO grade 4 | 0 (0.0%) | 0 (0.0%) | 0 (0.0%) | 0 (0.0%) |
| **Median Ki-67 (%)** | 12.5 (5.75, 30)^†^ | 2 (1.5, 26)^†^ | 4 (3, 10) | 80 |

Data are presented with n (%), Mean ± SD or Median (IQR). Some categorial variables do not add up to 100% due to missing values. Incomplete continuous variables are marked with †, all of which have ≤2 missing values except 5 in the “tumor maximum diameter” of “Diffuse pediatric-type high-grade glioma, H3-wildtype and IDH-wildtype”.

a. Symptoms of intracranial hypertension referred to headache and/or vomiting.

b. One patient only received bevacizumab and semustine.

Abbreviation: Amp, amplification; Non-amp, non-amplification; BMI, body mass index; KPS, Karnofsky Performance Score; TMZ, temozolomide.
